# Supplementary material for: H-Dot Mediated Nanotherapeutics Mitigate Systemic Toxicity of Platinum-Based Anticancer Drugs
Source: Int J Mol Sci. 2023 Oct 23;24(20):15466. doi: 10.3390/ijms242015466 (PMC10607179; doi:10.3390/ijms242015466)
Supplement: Supplementary file 1 [file ijms-24-15466-s001.zip › ijms-2662006-supplementary.pdf]

Supplementary Information

H-Dot Mediated Nanotherapeutics Mitigate Systemic Toxicity of Platinum-Based Anticancer Drugs

Supplementary Figures

Figure S1. UV absorption spectra of carboplatin with different concentrations.
Figure S2: SEC-HPLC result of Car/H-dot and H-dot.
Figure S3: NIT-1 cells treated with Car, H-dot, and Car/H-dot.
Figure S4: NIH3T3 cells treated with Car, H-dot, and Car/H-dot.
Figure S5: HEK293 cells treated with Car, H-dot, and Car/H-dot.
Figure S6: Organ weights of insulinoma mice after treatment with Car and Car/H-dot.
Figure S7: In vivo toxicity test at 7 days post-single injection in CD-1 mice treated with Car and Car/H-dot.

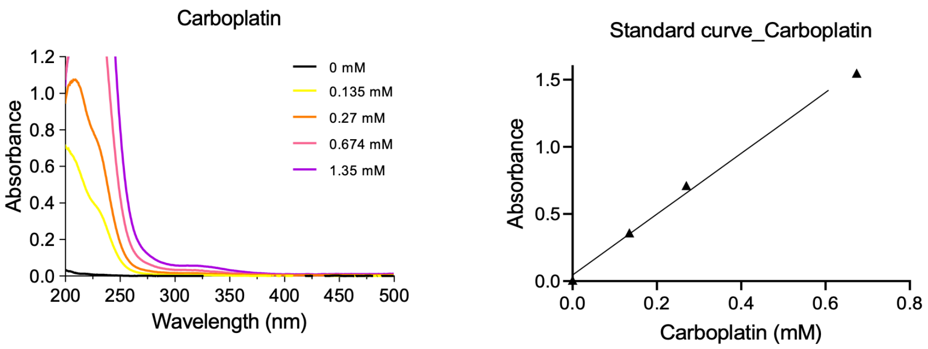

Supplementary Figure S1: UV absorption spectra of carboplatin with different concentrations. The standard curve of carboplatin was created with absorption values at 230 nm.

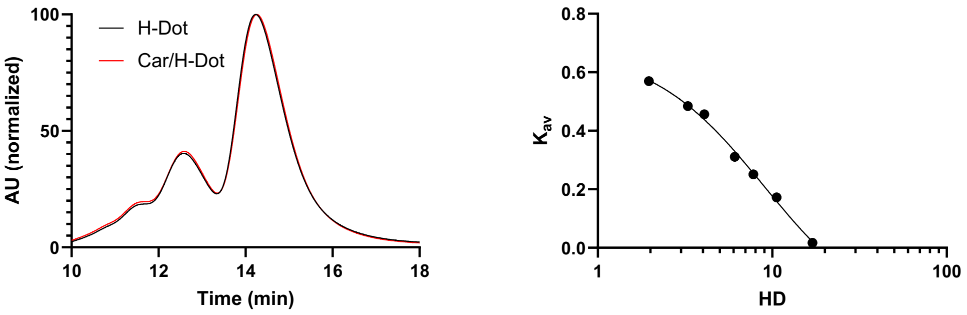

Supplementary Figure S2: SEC-HPLC result of Car/H-dot and H-dot. The standard curves were created by several proteins with varied molecular weights.

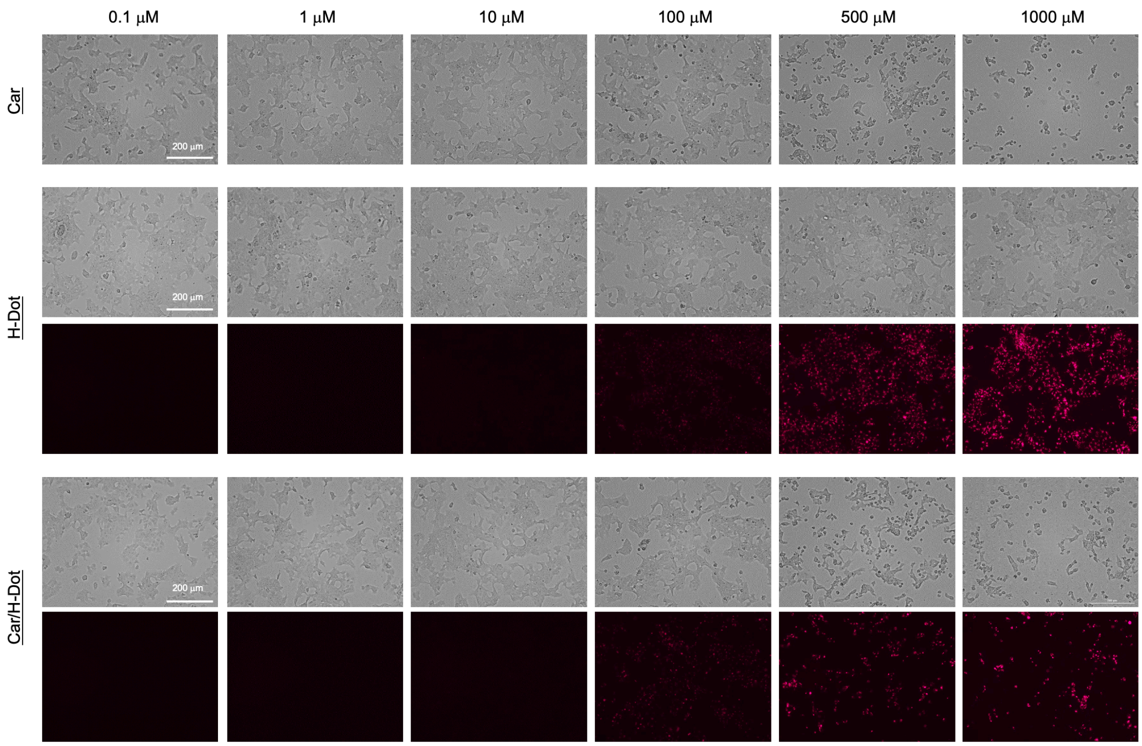

Supplementary Figure S3: NIT-1 cells treated with Car, H-dot, and Car/H-dot.

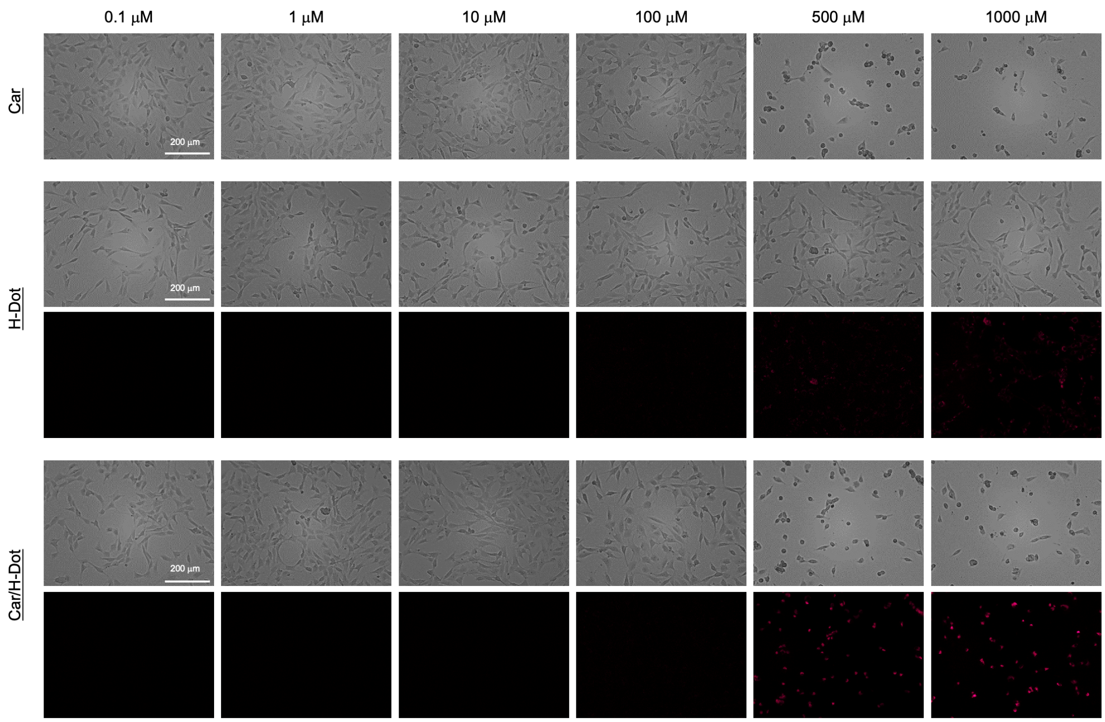

Supplementary Figure S4: NIH3T3 cells treated with Car, H-dot, and Car/H-dot.

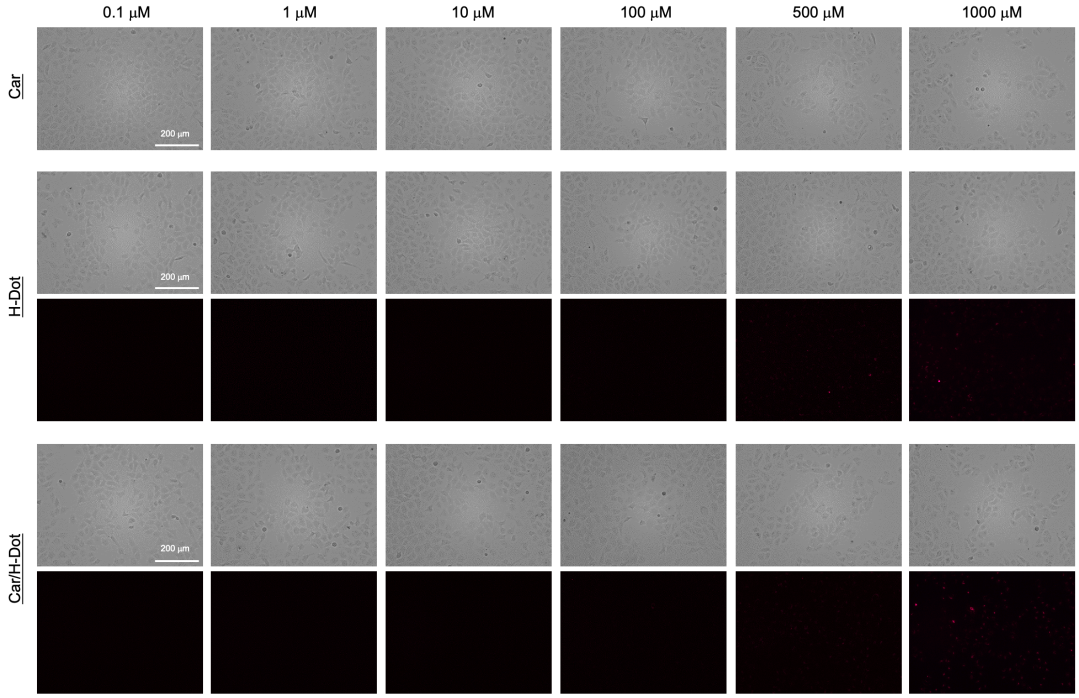

Supplementary Figure S5: HEK293 cells treated with Car, H-dot, and Car/H-dot.

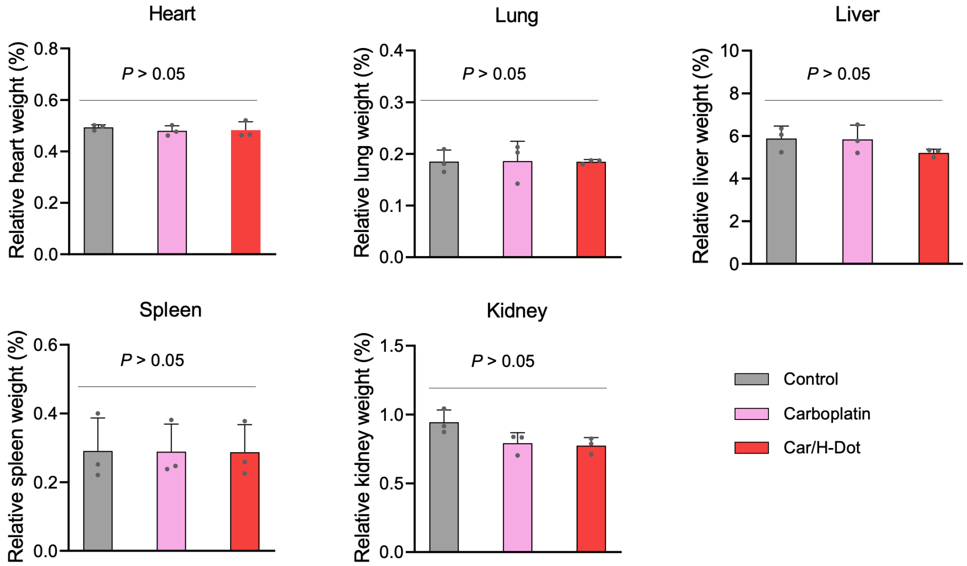

Supplementary Figure S6: Organ weights of insulinoma mice after treatment with Car and Car/H-dot. Saline was used as control. Each organ (heart, lung, liver, spleen, and kidney) was resected, and measure their weight at day 21 (n= 3 per each group).

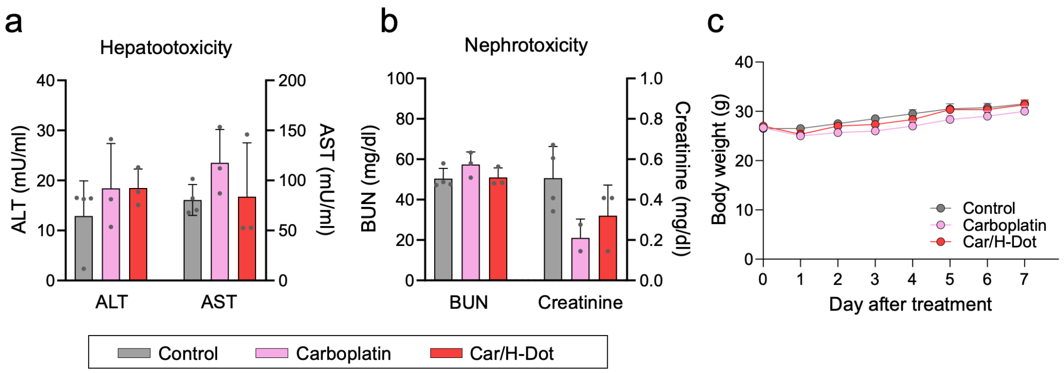

Supplementary Figure S7: In vivo toxicity test at 7 days post-single injection in CD-1 mice treated with Car and Car/H-dot (n= 3 per each group). Saline was used as control. (a) Serum aspartate transferase (AST) and alanine transferase (ALT). (b) Blood urea nitrogen (BUN) and creatinine. (c and d) Body weights of mice during post-7 days injection.
